# Outcomes following first-episode psychosis – Why we should intervene early in all ages, not only in youth

**DOI:** 10.1177/0004867416673454

**Published:** 2016-11-01

**Authors:** Julia M Lappin, Margaret Heslin, Peter B Jones, Gillian A Doody, Ulrich A Reininghaus, Arsime Demjaha, Timothy Croudace, Thomas Jamieson-Craig, Kim Donoghue, Ben Lomas, Paul Fearon, Robin M Murray, Paola Dazzan, Craig Morgan

**Affiliations:** 1 School of Psychiatry, University of New South Wales, Sydney, NSW, Australia; 2 Institute of Psychiatry, Psychology & Neuroscience, King’s College London, London, UK; 3 Department of Psychiatry, University of Cambridge, Cambridge, UK; 4 Department of Psychiatry, The University of Nottingham, Nottingham, UK; 5 School of Nursing and Health Sciences University of Dundee, Dundee, UK; 6 Nottingham University Hospitals NHS Trust, The University of Nottingham, Nottingham, UK; 7 Department of Psychiatry, Trinity College Dublin, Dublin, Ireland

**Keywords:** Psychosis, schizophrenia, early intervention, outcomes, women

## Abstract

**Objective:**

To compare baseline demographics and 10-year outcomes of a first-episode psychosis patient incidence cohort in order to establish whether current youth-focussed age-based criteria for early intervention services are justified by patient needs. The patients in this cohort were treated prior to the establishment of early intervention services. The study aimed to test the hypothesis that those who develop psychosis at a younger age have worse outcomes than those who develop psychosis at an older age.

**Methods:**

Data on first-episode psychosis patients from the ÆSOP-10 longitudinal follow-up study were used to compare baseline characteristics, and 10-year clinical, functional and service use outcomes between those patients who would and would not have met age-based criteria for early intervention services, in Australia or in the United Kingdom.

**Results:**

In total, 58% men and 71% women with first-episode psychosis were too old to meet current Australian-early intervention age-entry criteria (χ^2^ = 9.1, *p* = 0.003), while 21% men and 34% women were too old for UK-early intervention age-entry criteria (χ^2^ = 11.1, *p* = 0.001). The 10-year clinical and functional outcomes did not differ significantly between groups by either Australian- or UK-early intervention age-entry criteria. Service use was significantly greater among the patients young enough to meet early intervention age-criteria (Australia: incidence rate ratio = 1.35 [1.19, 1.52], *p* < 0.001; United Kingdom: incidence rate ratio = 1.65 [1.41, 1.93], *p* < 0.001).

**Conclusion:**

Current early intervention services are gender- and age-inequitable. Large numbers of patients with first-episode psychosis will not receive early intervention care under current service provision. Illness outcomes at 10-years were no worse in first-episode psychosis patients who presented within the age range for whom early intervention has been prioritised, though these patients had greater service use. These data provide a rationale to consider extension of early intervention to all, rather than just to youth.

## Introduction

Specialist early intervention services (EISs) provide intensive support and management for younger individuals in the early years following their first psychotic illness. There is notable variability internationally in the upper age threshold selected for EI service provision: in Australia, services are typically offered up to age 25 (Galletly et al., 2016); in Singapore to age 40. In the United Kingdom, 35 years has been the recommended upper age cut-off for referrals (Department of Health [DoH], 2001), but recent National Institute for Health and Care Excellence (NICE) guidelines recommend that early intervention (EI) should be available to all, regardless of age (NICE, 2014).

Historically, EI services have been youth-focussed based on several principles: first, the zeitgeist that the majority of psychosis presents earlier in life. Second, prior to EIS development, evidence that there was delay among young people with emerging psychosis obtaining early treatment (Lincoln and McGorry, 1995) and, finally, the theory that those who develop psychosis at a younger age suffer greater long-term impairment because the illness interrupts their social, personal and scholastic/occupational development (DoH, 2001).

Using data from the UK ÆSOP-10 study – a longitudinal follow-up of an incidence cohort of first-episode psychosis (FEP) patients – this study examined first, the baseline characteristics of FEP individuals who would and would not meet current age-based criteria for EIS in Australia or in the United Kingdom. Second, it tested the question whether 10-year clinical, functional and service use outcomes were worse in those who develop FEP at an age young enough to meet criteria for EI provision. It is important to emphasise that the cohort studied was treated in an *era prior to the establishment of EI services*; thus, this is *not* an examination of the effectiveness of EI care. Rather, these analyses compare baseline characteristics and 10-year outcomes of all FEP patients in order to establish an evidence base for EI provision by testing the theory that those who develop psychosis at a younger age have worse outcomes than those who develop psychosis at an older age.

## Methods

### Setting

This paper is based on data from ÆSOP (Kirkbride et al., 2006) and ÆSOP-10 (Morgan et al., 2014), which are incidence and 10-year follow-up studies, respectively, of all individuals with an FEP presenting for the first time to specialist mental health services in defined catchment areas in the United Kingdom between 1997 and 1999. Recruitment of ÆSOP cases ended before EI services were established in these areas.

### Cases

Within tightly defined geographical areas in London and Nottingham, all cases with FEP (codes F20–29 and F30–33 in International Classification of Diseases, Tenth Edition [ICD-10] (World Health Organization [WHO], 1993)) who presented to specialist services were included in the incidence study. The Screening Schedule for Psychosis (Jablensky et al., 1992) was used to screen cases who presented to these services for eligibility and completed based on information from clinical notes, corroboration from mental health staff and, where possible, by interview with the participant.

Inclusion criteria for cases were aged between 16 and 64 years with an FEP and resident within the study catchment areas. Exclusion criteria were evidence of psychotic symptoms precipitated by an organic cause, transient psychotic symptoms resulting from acute intoxication as defined by ICD-10 (WHO, 1993), previous contacts with mental health services for psychosis, and moderate or severe learning difficulties, or an IQ of less than 50 (WHO, 1993).

### Follow-up

Cases were followed-up 10 years after first contact with mental health services (detailed in Morgan et al. (2014). At baseline, 532 incidence cases were identified. Of those, 387 had follow-up outcome data and thus made up the core analytic sample for outcome analyses (excluding those who had died, emigrated or been excluded, plus those who did not have useable information on clinical course and outcome for at least 8 years of follow-up). Within our analyses of outcomes, we excluded six further cases as they presented to services less than 3 weeks before EIS were launched in the London catchment area.

### Measures

*Baseline*. Clinical and demographic data were collected from clinical records and, where possible, from interview. A shortened version of the Schedules for Clinical Assessment in Neuropsychiatry (SCAN version 2; WHO, 1994) was used to assess symptom presence and severity. This was used in conjunction with other clinical information (excluding diagnosis) to assign ICD-10 (WHO, 1993) psychotic diagnoses within research-team consensus meetings. Diagnoses were made blind to ethnicity and diagnosis from the clinical notes, as soon as possible after first contact. The Personal and Psychiatric History Schedule (WHO, 1996) was used to determine duration of untreated psychosis (DUP), defined as the period from onset of psychosis to first contact with statutory mental health services. Onset of psychosis was defined as the presence for 1 week or more of psychotic symptoms, further detailed in Morgan et al. (2006).
*Follow-up*. The WHO Life Chart Schedule (Harrison et al., 2001; Sartorius et al., 1996; Susser et al., 2000) – designed to assess the long-term course of schizophrenia – was used to collate information at follow-up. It comprises four main areas: symptoms, treatment, residence and work. It was adapted to include additional information on service use, including use of prescribed medication over follow-up. Information was derived for the Life Chart from multiple sources: case notes, interviews with cases and informant information. Key variables have been defined previously elsewhere (Morgan et al., 2014). Course of illness was categorised as follows: remission within 6 months, episodic (no episode longer than 6 months duration), continuous (no remission longer than 6 months duration) or none of the above. Course type ‘none of the above’ refers to an intermediate illness course which was neither episodic not continuous; these individuals experienced both an episode that lasted longer than 6 months and a period of remission that lasted longer than 6 months during the follow-up.

The Life Chart was also used to record number of inpatient days, mental state at follow-up (in the last 30 days; psychotic or not psychotic), history of self-harm over follow-up, lifetime substance misuse (present/absent) and percentage of time employed over follow-up (dichotomised into under and over 25%). The Life Chart was presented at consensus meetings along with case note information so that decisions about all aspects of the Life Chart could be decided upon by consensus. The Global Assessment of Function (GAF) disability scale (Endicott et al., 1976) was used to assess function at follow-up; higher score indicates better level of general functioning. Treatment resistance was defined in line with modified Kane criteria for treatment resistance (Conley and Kelly, 2001).

### Ethics

Full ethical approval for all aspects of the follow-up was provided by the local research ethics committees in South East London and Nottingham. All researchers had substantive or honorary contracts with either the South London and Maudsley National Health Service (NHS) Foundation Trust or the Nottingham Healthcare NHS Trust, the primary participating service providers.

### Analyses

All data were analysed using STATA 11 (StataCorp, 2009). Age at first presentation to specialist services was used to assign subjects to FEP groups by age. Using chi-square tests, we compared clinical and sociodemographic characteristics between those ⩽35 years and those ⩾36 years (UK-EI age-criteria); these analyses were repeated for those ⩽25 years and those ⩾26 years (Australia-EI age-criteria).

We compared outcomes in those ⩽35 years and those ⩾36 years (UK-EI age-criteria) using, as appropriate, logistic (binary or multinomial), Poisson or linear regression analyses. We adjusted analyses for gender, ethnicity, centre and diagnosis to assess whether variations in outcome by age group status were accounted for by these variables. Non-normally distributed continuous data were analysed using non-parametric bootstrap regressions (ordinary least squares). Bootstrap regressions were used as they produce the same coefficients as linear regression (and so are interpreted in the same way) but give more robust confidence intervals (CIs) and therefore a more robust estimate of statistical significance. Analyses were repeated forthose ⩽25 years and those ⩾26 years (Australia-EI age-criteria).

## Results

In total, 532 incidence cases were identified at baseline and comprised the sample for determining who would have been eligible for EI service provision. Demographic characteristics are detailed in Table 1. Table 2 shows the number and percentage of cases who would and would not have met age-entry criteria for EIS in Australia (⩽25 years) and in the United Kingdom (⩽35 years) by gender, DUP, diagnosis, treatment resistance and substance use.

**Table 1.** Sociodemographic and clinical characteristics of the entire sample (*n* = 532).

Characteristics	Number (%)
London	327 (61.5)
Nottingham	205 (38.5)
Male	308 (57.9)
Female	224 (42.1)
Schizophrenia broad	385 (72.4)
Manic psychosis	71 (13.4)
Depressive psychosis	76 (14.3)
White British	232 (43.6)
Other White	37 (7.0)
Black Caribbean	140 (26.3)
Black African	65 (12.2)
Asian	26 (4.9)
Other	32 (6.0)
Age at baseline (years), median (IQR)	29.0 (22.0–36.0)
DUP (days), median (IQR)	59.5 (15.0–235.0)
Follow-up (years), median (IQR)	10.4 (9.3–11.3)

IQR: interquartile range; DUP: duration of untreated psychosis

**Table 2.** Unadjusted analyses comparing characteristics in patients who would and would not have met age-entry criteria for EI services in the United Kingdom (⩽35 years or ⩾36 years) or in Australia (⩽25 years or ⩾26 years).

	Australia-EIS cut-off			UK-EIS cut-off		
	⩽25 years *n* (%)	⩾26 years *n* (%)	*p*	⩽35 years *n* (%)	⩾36 years *n* (%)	*p*
Gender (*n* = 532)						
Male	130 (66.3)	178 (53.0)	χ^2^ = 9.1, *df* = 1, *p* = 0.003	243 (62.1)	65 (46.1)	χ^2^ = 11.0, *df =* 1, *p* = 0.001
Female	66 (33.7)	158 (47.0)		148 (37.9)	76 (53.9)	
DUP (*n* = 532)						
DUP < 2 years	181 (92.4)	271 (80.7)	χ^2^ = 13.2, *df* =1, *p* < 0.001	346 (88.5)	106 (75.2)	χ^2^ = 14.4, *df* = 1, *p* < 0.001
DUP > 2 years	15 (7.7)	65 (19.4)		45 (11.5)	35 (24.8)	
Diagnosis (*n* = 532)						
Non-affective	147 (75.0)	238 (70.8)	χ^2^ = 5.8, *df* = 2, *p* = 0.056	287 (73.4)	98 (69.5)	χ^2^ = 11.5, *df* = 2, *p* = 0.003
Manic psychosis	30 (15.3)	41 (12.2)		59 (15.1)	12 (8.5)	
Depressive psychosis	19 (9.7)	57 (17.0)		45 (11.5)	31 (22.0)	
Treatment resistance (*n* = 257)						
Non treatment resistant	66 (69.5)	135 (83.3)	χ^2^ = 6.7, *df* = 1, *p* = 0.009	146 (74.1)	55 (91.7)	χ^2^ = 8.3, *df* = 1, *p* = 0.004
Treatment resistant	29 (30.5)	27 (16.7)		51 (25.9)	5 (8.3)	
Substance misuse (*n* = 419)						
No	102 (68.5)	222 (82.2)	χ^2^ = 10.4, *df = 1*, *p* = 0.001	222 (71.2)	102 (95.3)	χ^2^ = 26.6,*df* = 1, *p* < 0.001
Yes	47 (31.5)	48 (17.8)		90 (28.8)	5 (4.7)	

DUP: duration of untreated psychosis; EI: early intervention; EIS: early intervention service.

### Baseline illness profiles and characteristics

*Australia-EIS age-criteria*. Of 532 cases, 196 (36.8%) would have met age-entry criteria for EIS in Australia (Table 2). In all, 42% of men were ⩽25 years, compared with only 29% of women aged ⩽25. (χ^2^ = 9.1, df = 1,*p* =0.003) (Table 2). Thus, 58% of men and 71% of women were too old to meet Australian-early intervention age-entry criteria. There was a greater proportion ofpatients aged ⩾26 years who had a DUP > 2 years (19.4% compared to 7.7% in those ⩽25 years) (χ^2^ = 13.2,*df* = 1, *p* = 0.001). There was a non-significant trend for greater proportion of depressive psychoses in the 26+ years group (χ^2^ = 5.8, *df* = 2, *p* = 0.06). Treatment-resistant illness was significantly more common in the ⩽25 years group (30.5% compared to 16.7% in those ⩽26 years) (χ^2^ = 6.7, *df* =1, *p* = 0.009); as was substance misuse group (31.5% compared to 17.8% in those ⩾26 years) (χ^2^ = 10.4, *df* =1, *p* = 0.001) (Table 2).
*UK-EIS age-criteria*. Of 532 cases, 391 (73.5%)would have met age-entry criteria for EIS in the United Kingdom (χ^2^ = 11.0, *df* =1, *p* = 0.001) (Table 2). In all, 79% of men were ⩽35 years, compared with only 66% of women aged ⩽35 (χ^2^ = 11.0, *df* =1, *p* = 0.001). (Table 2). Thus, 21% of men and 34% of women were too old to meet UK-early intervention age-entry criteria. A total of 80 FEP cases presented with a DUP > 2 years and in some services would not have been accepted by EIS. Of those ⩽35 years, 11.5% had a DUP over 2 years, compared to 24.8% of those ⩾36 years (χ^2^ = 14.4, *df* =1, *p* < 0.001). Diagnostically, there were significantly higher proportions of manic psychoses in those ⩽35 years and of depressive psychoses inthose ⩾36 years (χ^2^ = 11.5, *df* =2, *p* = 0.003). There were significantly more cases of treatment-resistantillness (χ^2^ = 8.3, *df* =1, *p* = 0.004) and of substance misuse (χ^2^ = 26.56, *df* =1, *p* < 0.001) in those ⩽35 years (Table 2).

### Outcomes

The core analytic sample for analyses of outcomes at follow-up comprised 387 individuals (Morgan et al., 2014). For the analyses detailed here, six were excluded who presented less than 3 weeks before the introduction of EIS in the London catchment area for ÆSOP, giving a total sample of 381. Clinical, functional and service use outcomes are shown in Table 3 (Australia-based analyses) and Table 4 (UK-based analyses). Adjusted analyses have been included to provide information about how key variables (gender, centre, diagnosis and ethnicity) impact on outcomes; unadjusted analyses are reported because they reflect the service use and outcomes of the populations as they would bepresenting to EIS or other services, but it is important to note that for any given service, these outcomes would differ according to the demographics of the population being served.

**Table 3.** Differences in service use, clinical and functional outcomes between FEP patients who would and would not have met age-entry criteria for EI services in Australia (⩽25 years or ⩾26 years). Values in bold denote p < 0.05 in unadjusted analyses.

Outcome	⩽25 years	⩾26 years	Unadjusted analyses		Adjusted analyses^a^	
			Odds ratio (95% CI) (*unless otherwise indicated*)	*p*	Odds ratio (95% CI) (*unless otherwise indicated*)	*p*
Number of admissions (*n* = 334), median (IQR)	3 (1–6)	2 (1–4)	**1.35 [1.19, 1.52]^b^**	**<0.001**	1.33 [1.18, 1.51]^a^	<0.001
Proportion of follow-up days as inpatient (%)(*n* = 360), median (IQR)	3.0 (0.7–10.5)	1.9 (0.5–5.9)	**4.02 [1.51, 6.54]^c^**	**0.02**	3.61 [1.07, 6.15]^c^	0.005
Course of illness (*n* = 340)						
Continuous	27 (22.5)	51 (23.2)	–		–	
Remission within 6 months	13 (10.8)	29 (13.2)	0.85 [0.38, 1.89]	0.69	1.11 [0.48, 2.59]	0.80
Episodic	22 (18.3)	47 (21.4)	0.88 [0.44, 1.76]	0.73	1.03 [0.49, 2.16]	0.93
None of the above	58 (48.3)	93 (42.3)	1.18 [0.67, 2.08]	0.57	1.25 [0.68, 2.27]	0.47
Mental state at follow-up (*n* = 318)						
Non-psychotic	72 (65.4)	136 (65.4)	–		–	
Psychotic	38 (34.6)	72 (34.6)	1.00 [0.61, 1.62]	0.99	0.82 [0.49, 1.37]	0.44
Self-harm behaviour (*n* = 319)						
No	93 (82.3)	174 (84.5)	–		–	
Yes	20 (17.7)	32 (15.5)	1.17 [0.63, 2.16]	0.62	1.24 [0.66, 2.34]	0.51
Employment (*n* = 286)						
Employed less than 25% of follow-up	70 (69.3)	135 (73.0)	–		–	
Employed 25% or more of follow-up	31 (30.7)	50 (27.0)	1.20 [0.70, 2.04]	0.51	1.32 [0.75, 2.32]	0.33
GAF functioning (*n* = 282), mean (SD)	55.2 (17.6)	56.4 (18.6)	−1.19 [−5.63, 3.24]^c^	0.60	0.18 [−4.12, 4.34]^c^	0.94

IQR: interquartile range; CI: confidence interval; *p: p*-value; GAF: Global Assessment of Function; SD: standard deviation.

a Adjusted for baseline gender, centre, diagnosis and ethnicity.

b IRR: incidence rate ratio.

c Bootstrapped mean difference.

**Table 4.** Differences in service use, clinical and functional outcomes between FEP patients who would and would not have met age-entry criteria for EI services in the United Kingdom (⩽35 years or ⩾36 years). Values in bold denote p<0.05 in unadjusted analyses.

Outcome	⩽35 years	⩾36 years	Unadjusted analyses		Adjusted analyses^a^	
			Odds ratio (95% CI) (*unless otherwise indicated*)	*p*	Odds ratio (95% CI) (*unless otherwise indicated*)	*p*
Number of admissions (*n* = 334), median (IQR)	3 (1–5)	2 (1–3)	**1.65 [1.41, 1.93]^b^**	**<0.001**	1.59 [1.36, 1.86]^b^	<0.001
Proportion of follow-up days as inpatient (%)(*n* = 360), median (IQR)	2.6 (0.7–8.2)	1.2 (0.5–4.9)	**2.88 [0.54, 5.22]^c^**	**0.02**	1.80 [–0.61, 4.21]^b^	0.14
Course of illness (*n* = 340)						
Continuous	59 (23.3)	19 (21.8)	–		–	
Remission within 6 months	31 (12.3)	11 (12.6)	0.91 [0.38, 2.15]	0.83	1.30 [0.52, 3.28]	0.57
Episodic	50 (19.8)	19 (21.8)	0.85 [0.40, 1.77]	0.66	1.12 [0.51, 2.49]	0.78
None of the above	113 (44.7)	38 (43.7)	0.96 [0.51, 1.81]	0.89	1.07 [0.55, 2.05]	0.85
Mental state at follow-up (*n* = 318)						
Non-psychotic	151 (64.0)	57 (69.5)	–		–	
Psychotic	85 (36.0)	25 (30.5)	1.28 [0.75, 2.20]	0.37	1.06 [0.60, 1.88]	0.84
Self-harm behaviour (*n* = 319)						
No	194 (80.8)	73 (92.4)	–		–	
Yes	46 (19.2)	6 (7.59)	**2.88 [1.18, 7.04]**	**0.02**	3.37 [1.35, 8.49]	0.01
Employment (*n* = 286)						
Employed less than 25% of follow	151 (69.0)	54 (80.6)	–		–	
Employed 25% or more of follow	68 (31.1)	13 (19.4)	1.87 [0.96, 3.65]	0.07	2.16 [1.06, 4.37]	0.03
GAF functioning (*n* = 282), mean (SD)	55.5 (18.7)	57.3 (17.0)	−1.85 [−6.81, 3.11]^c^	0.46	0.07 [−4.19, 4.34]^c^	0.97

IQR: interquartile range; CI: confidence interval; *p* = *p*-value; FEP: first-episode psychosis; SD: standard deviation; GAF: Global Assessment of Function; EI: early invention.

a Adjusted for baseline gender, centre, diagnosis and ethnicity.

b IRR: incidence rate ratio.

c Bootstrapped mean difference.

#### Clinical outcomes

*Australia-EIS age-criteria.* There were no differences between groups in course of illness (OR = 1.18, 95% CI = [0.67, 2.08]), mental state at follow-up (OR = 1.00, 95% CI = [0.61, 1.62]) or self-harm (OR = 1.17, 95% CI = [0.63, 2.16]) (Table 3).
*UK-EIS age-criteria*. There was no difference between groups in course of illness over follow-up, with the highest proportion in both groups having a course that wasneither episodic nor continuous (OR = 0.96, 95% CI = [0.51, 1.81]), nor in mental state at follow-up (OR = 1.28, 95% CI = [0.75, 2.20]). More patients ⩽35 years engaged in self-harming behaviour over follow-up than those ⩾36 years (OR = 2.88, 95% CI = [1.18, 7.04] (Table 4).

#### Functional outcomes

*Australia-EIS age-criteria*. Neither GAF disability at follow-up (bootstrapped mean difference [BMD] = −1.19, 95% CI = [−5.63, 3.24]) nor employment over follow-up (OR = 1.20, 95% CI = [0.70, –2.04]) significantly differed between groups (Table 3).
*UK-EIS age-criteria*. Again, neither GAF disability atfollow-up (BMD = −1.85, 95% CI = [−6.81, 3.11]) nor employment over follow-up (OR = 1.87, 95% CI = [0.96, 3.65]) significantly differed between groups (Table 4).

#### Service use

*Australia-EIS age-criteria*. There was a higher number of admissions (incidence rate ratio [IRR] = 1.35, 95% CI = [1.19, 1.52], *p* < 0.001) and greater proportion of follow-up in hospital in those ⩽25 years (BMD = 4.02%, 95% CI = [1.51, 6.54], *p* = 0.02) (Table 3).
*UK-EIS age-criteria*. Those ⩽35 years were admitted to hospital over 10-year follow-up more often than those ⩾36 years (IRR = 1.65, 95% CI = [1.41, 1.93], *p* < 0.001). Proportion of follow-up spent in hospital was higher in those ⩽35 years (BMD = 2.88%, 95% CI = [0.54, 5.22], *p* = 0.02) (Table 4).

## Discussion

This study uses data from a large observational cohort of FEP patients to explore the evidence base for prioritisation of EI service provision in FEP. The first striking finding is that current EIS are gender-inequitable: more than two-thirds of women in Australia, and one-third of women in the United Kingdom, are excluded from current EIS because they are too old. Evidence that men have an earlier onset of psychosis is well-replicated (Rabinowitz et al., 2006), and 20% of women with schizophrenia have illness onset after the 40th year of life (Riecher-Rossler, 2007). Yet, there has been an absence of acknowledgement that age cut-offs for EI services disproportionately negatively impact on women. This is concerning, given recent meta-analytic evidence that recovery rates in psychosis are similar in men and women (Jääskeläinen et al., 2013).

Based on this observational incidence cohort of FEP, current EIS provision would be available to only 36.8% of FEP patients in Australia and to 73.5% in the United Kingdom. These findings concur well with evidence that 55% of FEP patients in Australia present after the typical EI service upper age limit of 25 years (Selvendra et al., 2014). Similarly, Greenfield et al. (2016) recently described that following the extension of their UK-based EI service to age 65, 30% of subsequent referrals were aged over 35 years (Greenfield et al., 2016). This evidence challenges the presently held misapprehension that psychosis is an illness of young people. In fact, epidemiological evidence indicates that FEP presents across the age-span, and, that with increasing age, there are increased relative proportions of females affected (Hafner, 2003).

DUP of greater than 2 years was significantly *more* common in the patients too old to be accepted for EI care in either Australia or the United Kingdom. Because these data pre-date the establishment of EIS, this finding is not explained by successful earlier detection through interventions targeted at the younger age group. Clearly, it is not only young people with emerging psychosis who are delayed in obtaining early treatment (Lincoln and McGorry, 1995). These findings are concerning and suggest delayed help-seeking and/or less adept detection of psychosis in this older age group. While longer DUP is known to be associated with poorer outcomes, despite longer DUP being more common in the older group, outcomes were similar to those in the younger group. Various explanations are possible: the association between longer DUP and poorer outcomes at 1–2 years following FEP is of moderate effect size; studies over longer follow-up periods show mixed findings (Marshall et al., 2005). Furthermore, the greater proportion of individuals with treatment-resistant illness (with associated poorer outcomes) in the younger age group may explain the lack of difference in outcomes between groups, despite the difference in DUP.

### Clinical and functional outcomes

There was no evidence that younger patients experienced poorer clinical outcomes: neither illness course nor likelihood of being psychotic at 10-year follow-up differed between those who were or were not below the age cut-offs. Nor was there any support for the hypothesis thatpatients presenting younger had poorer functional outcomes: neither employment nor disability (measured by GAF score) – differed between those who would and would not have met criteria for EIS in either Australia or United Kingdom. These findings indicate that older clients’ clinical and functional needs are at least as great as those of younger clients.

### Similar outcomes; different service use

Individuals young enough to meet current criteria for EIS in Australia or in the United Kingdom had greater service use: patients aged ⩽25 years spent proportionally longer in hospital during 10-year follow-up and were admitted at a greater rate: number of admissions over time was 35% greater than in those ⩾26 years. This may in part be explained by markedly higher rates of comorbid substance use and significantly higher rates of self-harm in the younger FEP group. Additionally, treatment-resistantillness was significantly more prevalent in the younger FEP group, possibly contributing to the observed higherservice use. Yet despite this greater service use, there was no difference in the clinical and functional outcomes at follow-up.

### Strengths and limitations

Strengths of this study include the epidemiologically robust methods employed in ÆSOP-10, previous studies of long-term course and outcomes have focussed on prevalence samples of patients with schizophrenia only (rather than all psychosis), which tend to have an over-representation of patients with poorer outcomes. Such studies have often applied an upper age cut-off well below 65 years (reviewed in Morgan et al., 2014), which may artificially perpetuate the idea that FEP is an illness only of young people. In ÆSOP-10, clinical and functional outcomes were considered separately. Approximately 40% of patients with a non-affective disorder achieved symptomatic recovery (Morgan et al., 2014), in accordance with the rate of 47% reported by Robinson and coworkers in their 5-year follow-up of first-episode schizophrenia patients (Robinson et al., 2004). Functional recovery was left often achieved: only 22% of ÆSOP-10 clients were employed at 10-year follow-up (Morgan et al., 2014). These recovery rates are in keeping with those reported elsewhere: Jääskeläinen and coworkers’ meta-analysis of clinical and social recovery rates in psychosis reported median rates of 13.5, with a range from 8% to 20% (Jääskeläinen et al., 2013).

Several limitations of this study should be acknowledged: first, as with all long-term follow-up studies, it is possible that selection or information bias might occur as a result of loss to follow-up/missing data. As detailed in Morgan et al. (2014), exhaustive tracing efforts resulted in follow-up of over 90% of the cohort, and analyses showed that there was no strong evidence of systematic bias. Second, the inferences made about the needs of patients of future EIS extend only to the age group of patients included here: 16–64 years.

## Conclusion

EIS have historically been youth-focussed, based on the premise that psychotic illness interferes at a key stage in a young person’s development. We do not dispute that premise, but emphasise that psychosis impacts at all stages of life. Interestingly, the use of the term EI in psychosis has for many come to be synonymous with intervention in *youth* psychosis: the idea of intervening *early* should not be conflated with intervening *in the young*.

We recommend that consideration be given internationally to the extension of EIS provision to all on the basis of clinical need and gender- and age-equality. A significant proportion of people suffering psychotic illness for the first time are currently exempt from specialist services. Discussion is warranted about the potential to deliver EI to all. Possible service models include EIS which open to all ages, with a recognition that care offered will need to be tailored to different needs of different age groups. Alternatively, in Australia, where there has been recent growth in youth-focussed mental health services, EI in psychosis could be managed both within these youth services and additionally in a dedicated service for psychosis in adults over 25 years. This would allow EI services to retain their youth focus, while also making necessary provision for older FEP patients with the same need for EI to optimise clinical and functional outcomes.
